# Survival Mechanisms of Metastatic Melanoma Cells: The Link between Glucocorticoids and the Nrf2-Dependent Antioxidant Defense System

**DOI:** 10.3390/cells12030418

**Published:** 2023-01-26

**Authors:** Elena Obrador, Rosario Salvador-Palmer, Rafael López-Blanch, María Oriol-Caballo, Paz Moreno-Murciano, José M. Estrela

**Affiliations:** 1 Cell Pathophysiology Unit (UFC), Department of Physiology, Faculty of Medicine and Odontology, University of Valencia, 46010 Valencia, Spain; 2 Scientia BioTech S.L., 46002 Valencia, Spain; 3 Department of Physiology, Faculty of Pharmacy, University of Valencia, 46100 Burjassot, Spain

**Keywords:** melanoma, stress, glucocorticoids, Nrf2, antioxidant defense

## Abstract

Circulating glucocorticoids increase during stress. Chronic stress, characterized by a sustained increase in serum levels of cortisol, has been associated in different cases with an increased risk of cancer and a worse prognosis. Glucocorticoids can promote gluconeogenesis, mobilization of amino acids, fat breakdown, and impair the body’s immune response. Therefore, conditions that may favor cancer growth and the acquisition of radio- and chemo-resistance. We found that glucocorticoid receptor knockdown diminishes the antioxidant protection of murine B16-F10 (highly metastatic) melanoma cells, thus leading to a drastic decrease in their survival during interaction with the vascular endothelium. The BRAF^V600E^ mutation is the most commonly observed in melanoma patients. Recent studies revealed that VMF/PLX40-32 (vemurafenib, a selective inhibitor of mutant BRAF^V600E^) increases mitochondrial respiration and reactive oxygen species (ROS) production in BRAF^V600E^ human melanoma cell lines. Early-stage cancer cells lacking Nrf2 generate high ROS levels and exhibit a senescence-like growth arrest. Thus, it is likely that a glucocorticoid receptor antagonist (RU486) could increase the efficacy of BRAF-related therapy in BRAF^V600E^-mutated melanoma. In fact, during early progression of skin melanoma metastases, RU486 and VMF induced metastases regression. However, treatment at an advanced stage of growth found resistance to RU486 and VMF. This resistance was mechanistically linked to overexpression of proteins of the Bcl-2 family (Bcl-xL and Mcl-1 in different human models). Moreover, melanoma resistance was decreased if AKT and NF-κB signaling pathways were blocked. These findings highlight mechanisms by which metastatic melanoma cells adapt to survive and could help in the development of most effective therapeutic strategies.

## 1. Introduction

Stressful events may precede cancer and stress-related psychosocial factors appear associated with higher cancer incidence and poorer survival [1]. The question of whether there is a link between stress and cancer has confused and interested both researchers and patients. Study after study has asked whether people who develop cancer have experienced more stress in the years before diagnosis and, conversely, if people who have experienced extreme stress are more likely to develop cancer. In this regard, epidemiological evidence continues to accumulate on the effect of psychosocial, behavioral and physic stress in relation to cancer risk, progression, and mortality [2,3,4]. Consequently, stress management appears essential for cancer patients, and particularly in the case of melanoma, a pathology in which there is a narrow barrier between benign lesions, malignant transformation, and metastatic spread [5].

Stress-induced diseases are the consequence of an excessive adrenergic response and mainly due to a glucocorticoid-dependent deterioration of the immune system. Stress is linked to a lower efficiency of natural cell repair processes [6], and emerging evidence suggest that DNA damage is increased by exposure to psychological stress and stress hormones [7]. Nevertheless, the key question is whether stress mediators may have a direct impact on DNA mutations, repair mechanisms, epigenetic changes, cancer spread and metastasis. In this sense, results from recent research are mixed since some experts suggest that stress can cause cancer, while others believe it may only contribute to the condition, see, e.g., [2,8].

Recent studies indicating that stress could facilitate cancer growth and even metastatic spread are mainly animal studies [3]. As pointed out by Eckerling et al. [3], “the stress response can facilitate cancer growth and metastasis through a direct action on the molecular characteristics of malignant tissue, on its microenvironment, on antitumor immune activity, and on other indirect modulators of progression”. For instance, cortisol increases the expression of the HPV16 E6 and E7 oncogenes, which facilitate degradation of p53 and, thereby, tumor initiation [9]. Moreover, stress conditions and/or increased levels of cortisol are associated to poor/bad nutrition, poor sleep and vitamin D deficiency, all factors that may favor the development of cancer [1].

In colon and gynecological cancers (endometrial, ovarian and triple negative breast cancers), it has been demonstrated that high glucocorticoid expression or glucocorticoid receptor (GR) activation are linked with cancer progression, development of treatment resistance, and/or a poorer patient prognosis [10,11,12,13,14,15]. Moreover, overexpression of GR induces cisplatin resistance through p38 MAP kinase in cervical cancer patients [16]. Prolonged serum elevation of glucocorticoid levels can negatively influence mitochondrial function leading to mitochondrial damage with a negative impact on cellular metabolism [17]. In addition, stress can reduce the body’s resistance to some types of viruses, which are now known to be significantly involved in the initiation of around 15% of cancer cases. Human papillomavirus [18], Epstein-Barr virus [19], Kaposi sarcoma-associated herpesvirus [20], and hepatitis C and B viruses [21] can be reactivated by catecholamines and glucocorticoids (e.g., [22,23]). Glucocorticoids are among the most potent immunosuppressive agents and, thus, may favor the progression of cancer [24]. They inhibit the synthesis of almost all known cytokines and of several cell surface molecules required for immune function [25]. Around 15–20% of all cancer cases are preceded by infection or chronic inflammation at the same organ site and, in many cases, inflammation exists long before tumor formation [26]. All these facts suggest that stress and glucocorticoids, in particular, could be involved in the initiation of the carcinogenic process, and favor the transformation of a physiological microenvironment into a protumoral milieu.

Nevertheless, moving from the level of animal research to human clinical research, both epidemiological studies and clinical trials have generated somewhat uncertain results, indicating only a small, not necessarily significant, effect of stress on cancer progression [3,27,28] (www.clinicaltrials.gov accessed on 5 January 2023). Consequently, the current medical routine does not include specific measures designed to prevent stress responses as a means of improving cancer survival [3]. Nevertheless, it seems reasonable to suggest that stress management interventions should be tested in the critical periods that affect cancer progression, especially in the short postoperative period and adjuvant treatments. In addition, more experimental studies will be needed to assess the long-term effects of treatments.

## 2. Melanoma Incidence, Prognosis and Therapeutic Challenges

Skin melanoma accounted for 4% of all new cancer diagnoses in EU-27 countries in 2020 (all cancers, excluding non-melanoma skin cancers) and for 1.3% of all deaths due to cancer (https://ecis.jrc.ec.europa.eu accessed on 3 January 2023). Ultraviolet (UV) solar radiation is the main source of skin damage that can lead to skin cancer. Both UV radiation and stress cause the body to produce reactive oxygen species (ROS) [29]. Those can increase inflammation and damage our skin’s DNA, leading to mutations and, possibly, skin cancer [29,30]. In fact, coupled with genetic and environmental factors, inflammation and stress have been suggested to play a role in melanoma formation and progression [31,32].

As in other tumors, the best prognosis is achieved by early removal of tumors. The two most common types of melanomas are [33]:

(a) Superficial spreading melanoma: this type accounts for 70% of melanomas and most often affects the legs of women and the torsos of men. Tumor cells usually have mutations in the BRAF gene.
(b) Nodular melanoma: 15 to 30% of melanomas, appears anywhere on the body, and grows rapidly.

The shallower the melanoma is in the skin, the greater the chance that surgery will cure it. Almost 100% of shallow and newer melanomas are cured by surgery. However, melanomas deeper than about 1 mm in the skin have a higher risk of metastasis to lymph nodes and spread through the blood. Once the melanoma has reached the lymph nodes, the 5-year survival rate varies between 25% and 70%. Once melanoma has metastasized to distant parts of the body, the 5-year survival rate is about 10% [34].

If the melanoma has spread to distant areas, surgery is not usually an option. Chemotherapy, such as dacarbazine and temozolomide, can be given intravenously to treat melanomas that have spread, but they still do not prolong survival and are given to people who have no other options. Radiation therapy may be used when the cancer has metastatized the brain (www.cancer.org accessed on 15 December 2022)

BRAF (vemurafenib, dabrafenib and encorafenib) and MEK (trametinib, cobimetinib and binimetinib) inhibitors have proven to improve survival in BRAF-mutant unresectable or metastatic melanoma [35], although half of the patients develop resistance within a year [36]. As the concurrent inhibition of the BRAF and MEK proteins could decrease MAPK-acquired resistance, and lead to a longer duration of response and overall survival, BRAF/MEK inhibitors are currently used in combinations in the clinical practice [37].

PD1, PD-L1, CTLA-4 and LAG-3 are immune checkpoint proteins physiologically expressed by immunocompetent cells to maintain immunological homeostasis and prevent autoimmunity. However, they can be used by cancer cells to down-regulate antitumor responses and evade the immune response [38]. Melanoma was the first malignancy to be treated with immunological checkpoint inhibitors (ICIs) [39]. ICIs are monoclonal antibodies that selectively bind to these proteins and reestablish the anti-tumor immune responses. Four classes of ICIs are approved by the FDA for the treatment of melanoma: ipilimumab (an antagonist of the cytotoxic-T lymphocytes antigen 4, CTLA-4) [40]; nivolumab and pembrolizumab, antagonists of programmed cell death protein 1 (PD-1) [41]; atezolizumab, an antagonist of programmed cell death ligand 1 (PD-L1) [42]; and relatlimab-rmbw, (a combination of the LAG-3-blocking antibody relatlimab and the programmed death receptor 1-blocking antibody nivolumab) [42]. Despite the increase in survival associated with the introduction of ICIs therapies [43], approximately half of the patients with melanoma do not obtain a lasting benefit [44,45].

In addition, over 350 NCI-registered clinical trials are currently being conducted. Many trials have been carried out to treat advanced or metastatic melanoma by using monotherapy or combination therapy of chemotherapy, immunotherapy, radiotherapy, and targeted therapy, and also new dosage forms or delivery systems (see [46]).

## 3. Intracellular Redox State and Oxidative Stress in Melanoma Initiation and Progression

Oxidative stress associates to an excessive ROS production, byproducts of O_2_ metabolism which also have key roles in cell signaling [47]. ROS-induced molecular damages and signaling activation of specific pathways can affect carcinogenesis and tumor progression [48,49]. Oxidative stress and/or redox status alterations may lead to cell transitions from quiescent to proliferative status, growth arrest and/or cell death depending on the importance of the redox imbalance [50]. Therefore, although oxidative stress and redox status shifts can cause cancer cell death, it is also feasible that they help to generate cell subsets capable of adapting and survive.

In addition to ROS, other reactive species can have a significant impact on the intracellular redox status, i.e., reactive nitrogen species (RNS), reactive sulfur species, reactive carbonyl species, reactive selenium species, chlorine and bromine species, also prooxidants such as transition metals (e.g., Mg^2+^, Cu^2+^ or Fe^2+^) or vitamins (e.g., Vitamin C), and some chemotherapeutic drugs (e.g., adriamycin and other anthracyclines, bleomycin, and cisplatin which bind to DNA and generate ROS, or quinones, highly redox active molecules which can cycle with their semiquinone radicals, leading to formation of ROS) [29].

Cancer cells, including melanoma cells, overproduce ROS compared to their normal cell counterparts [51,52,53]. ROS can be generated by mitochondria, the melanosomes, NADPH oxidase family enzymes, different arachidonic acid oxygenase activities, and the nitric oxide synthase activities [54]. In addition, an increased metabolism, as compared to normal melanocytes, interaction with immune and endothelial cells, UV radiation, and changes in the antioxidant system are factors that must be also taken into account to evaluate ROS levels and their effects on the growing melanoma [29]. For instance, H_2_O_2_ production is higher in melanoma cells than in melanocytes, and H_2_O_2_ induces higher tyrosinase activity (the rate-limiting enzyme in melanin synthesis) [55]. Moreover, melanin synthesis associates with higher levels of ROS, which turns the melanin/ROS ratio into a vicious cycle that favors the progression of melanoma [56]. Melanin, which is usually in an antioxidative reduced state within the melanosome, evolves during the pathogenesis of melanoma into a pro-oxidant substance that generates superoxide anion [57,58]. The important role of the antioxidant response during melanomagenesis is suggested by the overexpression of heme-oxygenase-1 (HO-1), a Nrf2 target. HO-1 is upregulated in B16-F10 murine melanoma cells and in different melanoma tumor models growing in vivo. Cells overexpressing HO-1, compared to controls, had increased proliferation rate, improved resistance to H_2_O_2_-induced oxidative stress, higher angiogenic activity, augmented metastatic activity, and decreased survival [59].

Cancer cells face replication stress caused by ROS-induced DNA damage, by oncogenic stress associated to dysregulation of fork progression, or by chemotherapy and radiotherapy. NOK-SI cells (human oral keratinocytes) stimulated with norepinephrine or cortisol showed higher DNA damage compared to untreated cells, whereas the hormone-induced DNA damage was reversed by pre-treatment with the β-adrenergic blocker propranolol [60].

Hence, there is much accumulated evidence of oxidative stress in melanoma cells growing in vitro and in murine models growing in vivo (e.g., [54,57,61,62,63,64,65,66]). Consequently, upregulation of their antioxidant defenses appears necessary to guarantee their survival, or at least that of the most resistant clones.

## 4. Stress Hormones and Melanoma Growth

As suggested by different studies and as pointed out by Sanzo et al. [67], chronic stress, involving environmental and psychological factors, could be a relevant cofactor in melanoma progression and spreading. In this, different risk factors have been suggested, i.e., excessive body mass index, high stress-related activities, or immunosuppression [68]. Moreover, stress hormones can cause upregulation of cytokines, i.e., VEGF, TGF, IL6 or IL8, which are proangiogenic and/or favor tumor progression [32,69]. Therefore, it seems plausible to infer that melanoma progression may be inhibited by blocking the molecular signaling cascades involving specific cytokines.

IL-6 is dysregulated in many types of cancers, and increased serum levels of IL-6 have been correlated with a worse prognosis in patients bearing different cancers, including melanoma [29,70]. In this regard, it has been shown that solid tumor cells may secrete high levels of IL-6, which is involved in fundamental processes in cancer metastasis, i.e., angiogenesis, proliferation, attachment, and invasion (e.g., [71,72]). In the classic B16-F10 melanoma model, known for its high metastatic potential, we observed that IL-6 (mainly derived from the melanoma cells) promotes the release of glutathione (GSH) from hepatocytes to the circulating blood [73]. This facilitates GSH to reach distant growing metastases. The plasma membrane-bound γ-glutamyl transpeptidase (GGT) enzyme degrades extracellular GSH, thus releasing cysteinyl-glycine (further metabolized by dipeptidases) and γ-glutamyl amino acids [74,75,76]. Free cysteine, glycine and γ-glutamyl-amino acids are taken up by the melanoma cells and can be used as GSH precursors [77]. Indeed, in the B16-F10 model, GGT activity and the interorgan transport of GSH promote the synthesis of GSH in the melanoma cells and their metastatic growth [78]. In this mechanism, the liver plays an essential role because it is the major physiological reservoir of GSH [79].

The neuroendocrine and immune systems work in order of maintaining homeostasis under conditions that favor overproduction of cytokines [80]. The hypothalamus-pituitary-adrenal (HPA) axis can be stimulated by cytokines (e.g., IL-1, IL-6, or αTNF), which are overproduced in many different immune, inflammatory or neoplastic processes [81]. Consequently, the HPA axis increases secretion of ACTH, thereby activating the synthesis and release of glucocorticoids from the adrenal glands [82]. Interestingly, pathophysiological concentrations of cortisol have been shown to increase IL-6 production by, e.g., human squamous cell carcinoma cells [83]. Moreover, in patients with advanced ovarian cancer, increased levels of IL-6 in ascitic fluid correlated with increased salivary cortisol [84]. More importantly, tumor-derived IL-6 impairs the ketogenic response to reduced caloric intake, thus promoting a systemic metabolic stress response that blocks anti-cancer immunotherapy [85]. Thus, suggesting a role of IL-6 to increase glucocorticoid secretion, and the consequent immune suppression. Facts that raise the question of whether glucocorticoids should be targeted in conjunction with immunotherapy interventions.

Glucocorticoids are useful in the primary combination chemotherapy of both acute and chronic lymphocytic leukemias, Hodgkin’s and non-Hodgkin’s lymphomas, multiple myeloma and breast cancer [86]. Glucocorticoids work through their receptors to perform a variety of functions, including arresting growth or inducing apoptosis in lymphocytes [86]. The glucocorticoid-induced apoptosis appears to involve multiple signaling pathways, i.e., transactivation of apoptosis inducing genes, such as Bim, and the negative modulation of survival cytokines through inhibition of AP-1 and NF-κB mediated transcriptions [87,88,89]. However, they seem to blunt different chemotherapeutics, as it occurs, e.g., in ovarian cancers [90] or in many other tumors [91]. Moreover, glucocorticoids are also likely to blunt immunotherapies by interfering with immune responses [92,93]. Thus, it seems reasonable to think that inhibition of the GRs may help to prevent these problems.

Furthermore, pathophysiological levels of noradrenaline favor overexpression of VEGF, IL-8, and IL-6 in different human melanoma cell lines, and cytokine production are progressively increased in the metastatic phenotypes [31]. β-adrenoceptors are upregulated in human melanoma and their activation releases pro-tumorigenic cytokines [94], whereas α-adrenoceptor stimulation appears to attenuate melanoma growth in mice [95]. Furthermore, catecholamines have been found to increase proliferation of murine melanoma B16-F10 cells [96]. Based on the results of retrospective and prospective observational studies, Giorgi et al. recently suggested that β-blockers treatments should be considered as a treatment in melanoma, although clinical trials would obviously be needed to test their efficacy [97].

Therefore, based on this background, it is plausible that glucocorticoids and catecholamines may influence melanoma growth and IL-6 production in its metastatic cells. Further work in the B16-F10 melanoma model showed that plasma levels of ACTH, corticosterone and noradrenaline increase in mice bearing B16-F10 lung or liver metastases, as compared to non-tumor-bearing controls [98]. Corticosterone and noradrenaline, at pathophysiological levels, increased expression and secretion of IL-6 in the B16-F10 cells, which involves changes in the DNA binding activity of NF-κB, cAMP response element-binding protein, AP-1, and nuclear factor for IL-6 [98]. Moreover, in vivo inoculation of B16-F10 cells transfected with anti-IL-6-siRNA, treatment with the GR blocker RU-486 or with propranolol (a β-adrenoceptor blocker), increased hepatic GSH whereas decreased plasma IL-6 levels and metastatic growth [98]. In addition, IL-6 may also promote mechanisms to avoid the stress- and/or cytotoxic drug-induced metastatic cell death (e.g., increased expression of several survival proteins, such as Bcl-2, Bcl-xL, Mcl-1, survivin, and XIAP) [98,99].

**Figure 1:**
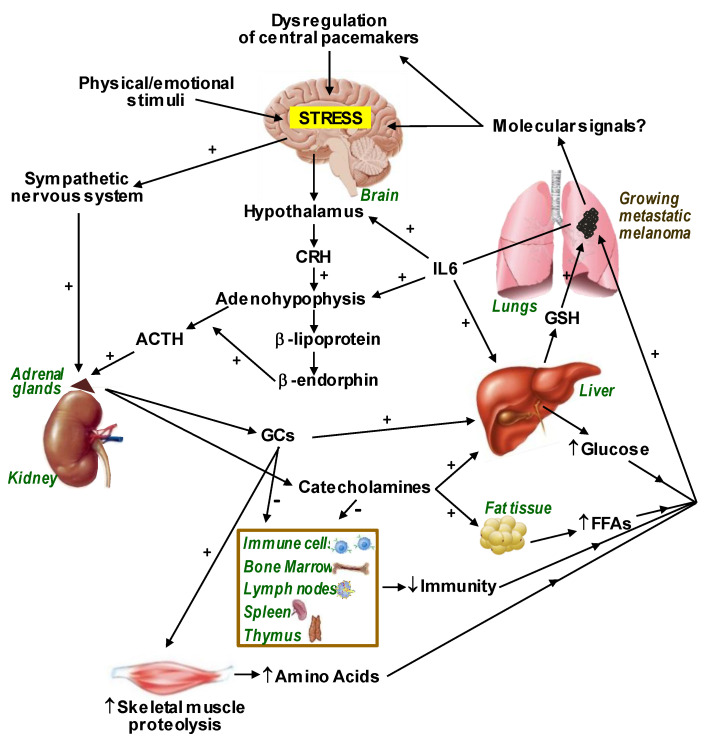
Pathophysiology of Stress in Metastatic Melanoma. Stress-induced dysregulation of central pacemakers and IL-6 (mainly from metastatic cells) favor the release of pituitary ACTH. IL-6 and catecholamines increase glutathione (GSH) release from the liver. Metastatic cell γ-glutamyl transpeptidase degrades plasma GSH, providing extra cysteine for GSH synthesis. GSH is a main physiological antioxidant involved in promoting metastases growth. Glucocorticoids (GCs) upregulate the Nrf2-dependent defense system in metastatic cells. Stress hormones decrease the immune response and facilitate the provision of nutrients to the growing metastases. Tissue-specific microenvironments or the influence of tumor innervation can also be decisive in the behavior of metastatic cells. ACTH, adrenocorticotropic hormone; CRH, corticotropin releasing hormone; AAs, amino acids, FFAs, free fatty acids.

Figure 1 schematically describes the pathophysiology of stress and its metabolic consequences in metastatic melanoma-bearing mammals.

## 5. Glucocorticoids and the Antioxidant Defense of Melanoma Cells

### 5.1. Glucocorticoids, Nrf2 and the Antioxidant Defense of Melanoma Cells

The transcription activator Nrf2 is the master regulator of the antioxidant response and upregulates the expression of antioxidant and detoxifying enzymes [100]. Nrf2 has a protective role in UV-induced oxidative stress, DNA damage, and apoptosis of melanocytes [101]. Given the important contribution of UV radiation for ROS formation, it is not surprising that Nrf2 activity is induced by UV in melanocytes [100]. Nrf1 and Nrf2 transcription factors, upon activation by oxidative stress, form heterodimers with different factors, i.e., Maf and Jun, to bind to the antioxidant/electrophile response element (ARE/EpRE) and regulate the transcription of oxidative stress/cytoprotection-related genes [59,102]. This is important because oncogene (i.e., KRAS, BRAF or MYC)-induced Nrf2 transcription activity associates to increases in melanoma growth and pharmacological resistance [103,104,105]. Elevated Nrf2 expression and a high GSH/GSSG ratio in melanoma are correlated with a deeper Breslow index, invasive/metastatic phenotype, and poor survival [106,107,108]. In that sense, it was shown that GSH protects melanoma from oxidative stress, contributing to its survival [107,109]. These results are in agreement with Beberok et al. who demonstrated that treatment with the antibiotic Lomefloxacin induce oxidative stress, GSH depletion and apoptosis in the COLO829 melanoma cell line [110]. Moreover, N-acetylcysteine (a classical mucolytic drug) can promote melanoma metastases spread, a fact suggesting that caution should be taken when administering GSH promoters to cancer patients [111]. Furthermore, the link between Nrf2 and immune tolerance has already been shown in lung adenocarcinoma, where Keap1 mutations are present in up to 20% and lead to permanent Nrf2 activation [112]. This is an important question in the case of metastatic melanomas where the anti-PD-1 immune therapy represent the only optional treatment [113,114].

Since glucocorticoids increase ROS generation in metastatic B16-F10 melanoma cells [98] and also in breast cancer cells [115], we investigated if the decrease in antioxidant enzyme activities in invasive B16-F10 cells (iB16) knockdown for the GR (iB16-shGR) was associated with changes in nuclear Nrf1 and/or Nrf2. Nuclear Nrf2, and not Nrf1, decreased in iB16-shGR cells isolated from lung or liver metastatic foci compared to control iB16 cells [116]. This is a fact that may be key since increased Nrf2 transcription activity has been correlated with aggressiveness in different human cancers [117,118,119]. However, other authors have found just opposite results postulating that GR signaling represses the antioxidant response, e.g., by inhibiting the histone acetylation mediated by Nrf2 [120], or by forming a glucocorticoid-GR complex that migrates to the nucleus where it binds to glucocorticoid response elements and ARE/EpRE sequences [121,122]. These controversial results should be analyzed in the light of the actual doses of glucocorticoids administered. Ligand-occupied GR induces or represses the transcription of thousands of genes by direct binding to DNA response elements and/or by physically associating with other transcription factors, thus involving a vast array of molecular interactions [123]. It is then essential to differentiate between pathophysiological and pharmacological levels, the latter being much higher (as reported in [120,122]) and potentially causing very different results. Indeed, biological stressors can positively or negatively affect antioxidant enzymes depending on the time and levels of exposure [124]. In fact, exposure to physiological stressors induces the production of ROS and oxidative stress in, e.g., the rat liver [125]. More importantly, a meta-analysis of glucocorticoids as modulators of oxidative stress concluded that glucocorticoids promote oxidative stress [126], which cannot be the result of improving the antioxidant defenses. Although, glucocorticoids are currently used against different cancers [127], they can also induce cancer resistance, a still unclear effect that may promote growth and metastases [127,128]. In fact, at pathophysiological levels, glucocorticoid signaling is antiapoptotic in cells of epithelial origin and in many malignant solid tumors subjected to cytotoxic therapy [89,129,130,131]. It was shown, in the immortalized human mammary epithelial cell line MCF10A, that GR-mediated protection from apoptosis is associated with induction of the serine/threonine survival kinase gene, sgk-1 [132]. In agreement with these ideas, GR antagonism has been shown to promote apoptosis in solid tumor cells [133].

**Figure 2:**
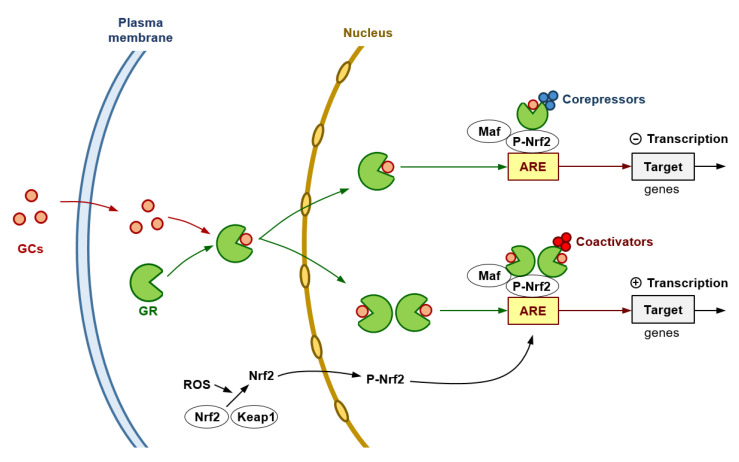
Glucocorticoids and the Antioxidant Defense of Melanoma Cells. The GR is encoded by the NRC31 gene which can produce a number of receptor isoforms, the GRα being the primary receptor involved in glucocorticoid (GC) signaling. The cytosolic GR complexes with different proteins, i.e., Hsp90, Hsp70 and the FK506-binding protein 4. GCs diffuse through the plasma membrane into the cytoplasm and binds to the GR resulting in the release of heat shock proteins. Based on the two-part model proposed by Gerber et al. [142], the cytoplasmic GR interacts with glucocorticoids, thus causing a conformational change and nuclear translocation. GR interacts with both the DNA and other transcriptional machinery to orchestrate its genomic effects through three main mechanisms: direct binding to glucocorticoid response elements, transcription factor tethering, and binding of composite elements within the DNA [143]. Hypothetically, at high pharmacological levels of GCs, primary repression could result from an excessive amount of GR monomers tethering to the ARE-Nrf2 complex (Nrf2 dimerizes with a basic region-leucine zipper bZIP protein and binds to the ARE to activate gene transcription), then leading to recruitment of corepressors. In this mechanism, GR associates with NF-κB or AP-1 [144,145], thus resulting in repression of their activity in a process attributed to recruitment of transcriptional corepressors such as the nuclear receptor co-repressor 1 and the histone deacetylase 2 [146]. At lower extracellular levels of GCs (pathophysiological levels), GR homodimers (predominantly formed) would recruit coactivators (such as the steroid receptor coactivator-1, SRC-1; or the GR-interacting protein-1, GRIP-1) [147] and induce the transcription of genes encoding antioxidant/defense enzyme activities. This double model may help to reconcile the controversy in the results obtained by different groups.

Importantly, at high levels or long-term exposure of ROS, p53 expression (promoted by DNA damage) increases, activating prooxidant genes, interfering with the Nrf2-dependent transcription of ARE/EpRE-containing promoters (and, thereby, inhibiting the Nrf2-mediated survival response), and potentially resulting in cell death [134]. However, particularly in highly aggressive human cancers, the p53 protein is reduced, lost, or mutated [135]. In this scenario, we used AS101 (ammonium tri-chloro(dioxoethylene-O,O′-)tellurate), a synthetic compound which has immunomodulating properties [136] and increases expression of wild-type p53 [137]. We observed that AS101-induced up-regulation of p53 in iB16 melanoma cells caused a decrease in antioxidant enzyme expression without affecting the nuclear levels of Nrf2 [116]. An effect that was reversed by using anti-p53 antisense oligonucleotides [116]. Thus, proving that p53 can suppress the Nrf2-dependent transcription of antioxidant enzymes in metastatic melanoma cells. Interestingly GR activation may lead to inhibition of p53-induced apoptosis, an effect observed in, e.g., MCF-10Amyc cells [138]. In agreement with this concept, in estrogen receptor-positive breast cancer cells, low GR expression has been linked to higher p53 expression [139]. Indeed, p53 can form a complex with the glucocorticoid that causes a cytoplasmic sequestration of both molecular structures [140]. These facts suggest a close link among GRs, p53 and Nrf2 which could be involved in regulating growth and spread of BRAF^V600E^-mutated melanoma cells. Figure 2 summarizes, as a working hypothesis, potential molecular interactions that may involve GRs and the Nrf2-dependent antioxidant defenses in melanoma cells.

Upon interaction of circulating melanoma cells with the vascular endothelium, a cascade of molecular events associates to the classical docking and rolling, i.e., attachment to the endothelial cells, release of proinflammatory cytokines, ROS and RNS, and tumor cytotoxicity [141]. Independently of the tumor location (liver, lung, or subcutaneous), in metastatic melanoma cells, GR knockdown decreased the expression and activities of γ-GCS, superoxide dismutase 1 and 2, catalase, glutathione peroxidase, and glutathione reductase, inducing a reduction in GSH levels [111]. Facts showing that GR knockdown compromises the antioxidant defense of melanoma cells, and increases the endothelium-induced tumor cytotoxicity [116]. Hence limiting their invasive capacity. This opens up potential therapeutic application in case selective GR blockers may show pharmacological efficacy under in vivo conditions.

### 5.2. Combined Glucocorticoid Receptor Antagonism and BRAF Inhibition Promotes Regression of Early Melanoma Metastases

Mifepristone (RU486) is a steroidal antiprogestogen (IC50 = 0.025 nM), as well as an antiglucocorticoid (IC50 = 2.2 nM), and antiandrogen (IC50 = 10 nM) to a much lesser extent [148]. Its relative binding affinity at the GR is more than three times that of dexamethasone and more than ten times that of cortisol [149]. The proposed mechanism of action of RU486 it is a competitive binding to the GR that prevents the dissociation of the heat shock proteins from the receptor avoiding its subsequent translocation to the nucleus and transcriptional activity [150]. RU486 does not bind to the estrogen receptor or the mineralocorticoid receptor [151]. Research work has revealed that progesterone can inhibit human melanoma cell growth. The mechanism of inhibition is due to autophagy and this effect of progesterone is not mediated through progesterone receptor [152]. Down-modulation or pharmacological inhibition of androgen receptors suppresses melanomagenesis, with increased intratumoral infiltration of macrophages and, in an immune-competent mouse model, cytotoxic T cells [153]. However, intracellular signaling derived from activation of progesterone or androgen receptors is different from that derived from GRs [154]. Recent studies have demonstrated cytotoxic and anti-metastatic effects of RU486 in vitro and in clinical trials involving meningioma, colon, breast, and ovarian cancers (e.g., Ritch et al. [155]), whereas Alvarez et al. [156] demonstrated that RU486 impedes the proliferation of uveal melanoma cells (a highly metastatic and drug resistant cancer). Furthermore, metapristone (the most active metabolite of RU486) inhibited cell viability and induced early and late apoptosis in B16-F10 and A375 melanoma cells [157]. Metapristone treatment resulted in decreased of Akt and ERK phosphorylation and of Bcl-2 and facilitated overexpression of p53 and Bax in A375 cells. In addition, metapristone suppressed cell migration and invasion by down-regulating the expression of matrix metalloproteinases (2 and 9), N-cadherin and vimentin, while E-cadherin expression was up-regulated [157].

The BRAF^V600E^ mutation is the most commonly observed in patients, represents more than 90% of BRAF mutations in melanoma, and can be detected early during melanoma development [158]. B-Raf signaling create a balance between a pro-oncogenic signal and a senescent proliferative arrest. Interestingly, in human fibroblasts BRAF^V600E^-induced senescence was bypassed by the addition of glucocorticoids (albeit at pharmacological doses), which allowed their cancer transformation [159].

Vemurafenib (VMF)/PLX40-32 (a selective inhibitor of mutant BRAF^V600E^) was the first molecularly targeted therapy to be licensed in the US and Europe for treatment of advanced melanoma. Its mechanism of action involves selective inhibition of the mutated BRAF V600E kinase that leads to reduced signaling through the aberrant mitogen-activated protein kinase pathway [160]. It has been reported that VMF increases mitochondrial respiration-linked ROS generation in BRAF^V600E^ melanoma cell lines [161]. However, VMF also induces HO-1 upregulation in primary BRAF^V600E^ melanoma cell lines, limiting the efficacy of the drug and reducing the cancer cell recognition and killing by natural killer cells [162]. Thus, possibly, a GR antagonist could increase the efficacy of BRAF-related therapy in BRAF^V600E^-mutated melanoma. To test this hypothesis, we studied the effect of RU486 [163] on the antioxidant defense of different human BRAF^V600E^ melanoma cell lines. We found that in vivo administration of RU486 to mice bearing metastatic BRAF^V600E^-mutated melanoma cells decreases Nrf2- and redox state-related enzyme activities and, in parallel, increases ROS production [109]. Further experiments showed that combined treatment with RU486 and VMF strongly inhibits BRAF^V600E^-mutated metastatic melanoma growth in vivo [109]. Importantly, melanoma growth inhibition was only observed if RU486 and VMF were administered simultaneously. However, if administration of VMF was delayed, the inhibitory effect of the association practically disappeared [109]. Thus, suggesting that, despite RU486 administration, melanoma cells can spontaneously develop anti-VMF resistance. Indeed, it is well known that the anti-melanoma effects of VMF are sort-lived, and that patients present tumor relapse in a short period after treatment [164,165]. In fact, melanoma cells showing acquired resistance to VMF have high rates of mitochondrial respiration associated with elevated mitochondrial oxidative stress [161]. Thus, suggesting that targeting the antioxidant defense could be the right therapeutic choice.

It is also worth to mention that the most common adverse effects of VMF treatment, i.e., pyrexia, arthralgia or skin rash, are usually treated with dexamethasone [166,167]. However, based on the above discussion, this therapy should be reconsidered as it has been recently recommended by the Oncological Endocrinology research group of the Italian Society of Endocrinology [168].

### 5.3. Anti-Death Adaptations Related to the Bcl-2 Family of Proteins in Advanced BRAFV600E-Mutated Melanoma Metastases

Recent research indicate that a stress-like state promotes overexpression of fos, hsp70 and ubb, all required for adaptation to diverse cellular stresses. This state has a higher tumor seeding capabilities compared to non-stressed cells, and confers intrinsic resistance to MEK inhibitors, commonly used in melanoma treatment [169]. Furthermore, this stress-like program can be induced by, e.g., heat shock, and promotes resistance to both MEK and BRAF inhibitors in human melanomas [169]. Further mechanisms of acquired melanoma resistance involve activation of the MAPK pathway. The PI3K-PTEN-AKT pathway is a 2nd resistance pathway, which often overlaps with the MAPK pathway [170,171]. Acquired resistance to MAPK pathway targeted therapies (BRAF/MEK inhibitors) develops in most patients at approx. 12 months [172]. Interestingly, it was also shown that GR-induced MAPK phosphatase-1 (MPK-1) expression inhibits paclitaxel-associated MAPK activation and contributes to breast cancer cell survival [173]. Moreover, the MEK/ERK signaling pathway regulates expression of different Bcl-2-related proteins and survival in, e.g., human pancreatic cancer cells [174,175]. We observed that different melanoma cells, surviving after RU486 treatment, down regulated expression of different Bcl-2-related pro-death genes (i.e., bax, bak, bid), whereas upregulated anti-death bcl-xl and mcl-1 [109]. Thus, we investigated if inhibition of Bcl-xL or Mcl-1 could improve the anti-melanoma effects of RU486 and VMF. Previously, dexamethasone was found to inhibit TRAIL-induced apoptosis of thyroid cancer cells via Bcl-xL induction [176]. Indeed, the treatment with RU486 + VMF + UMI-77 (UMI77 is a selective small-molecule inhibitor of Mcl-1 [177]) or RU486 + VMF + WEHI77 (WEHI77 is Bcl-xL-selective BH3 mimetic [178]) almost induced a complete regression of advanced BRAF^V600E^-mutated melanoma metastases depending on which of these Bcl-2-related proteins was preferentially overexpressed in the different human BRAF^V600E^ melanomas tested [109]. These findings are very relevant since melanoma regression was also associated to an increase in host survival [109], and because BRAF^V600E^ mutation can also be present in other malignant neoplasms such as hairy-cell leukemia, colon carcinoma, ovarian low-grade serous carcinoma, Langerhans cell histiocytosis and Erdheim-Chester disease, glial neoplasms and thyroid carcinoma [179].

## 6. Conclusions

To date, and based on the epidemiological studies carried out, there is insufficient evidence to establish a conclusive relationship between stress and the incidence/progression of melanomas. However, the experimental and clinical evidence mentioned and discussed in this review do make us suspect the existence of such a relationship. In this sense, and specifically regarding glucocorticoids, we may summarize the following facts: (a) although glucocorticoids are widely used in cancer therapy due to their proapoptotic properties in different tumor cells (i.e., acute and chronic lymphocytic leukemias, Hodgkin’s and non-Hodgkin’s lymphomas, multiple myeloma and breast cancer), they may also induce a yet undefined resistant phenotype which may facilitate tumor growth and metastases. In fact, different studies have demonstrated that glucocorticoids can suppress tumor progression, whereas other investigations reported that glucocorticoids inhibit chemotherapy-induced cancer cell death. This controversial phenomenon may result from different cancer subtypes, differential GR expression, different interactions at the transcription level and the dosage of glucocorticoid given; (b) glucocorticoids, at pathophysiological levels (non-pharmacological), can induce antiapoptotic signals which are associated with cancer resistance; (c) during early progression of skin melanoma metastases, RU486 (mifepristone, a GR antagonist) and VMF (vemurafenib, a BRAF inhibitor approved by the FDA for the treatment of late stage melanoma) induced a drastic metastases regression; (d) treatment at an advanced stage of growth is associated to the development of resistance to RU486 and VMF; (e) this resistance was mechanistically linked to overexpression of specific proteins of the Bcl-2 family (i.e., Bcl-xL and Mcl-1); (f) melanoma resistance was decreased if AKT and NF-κB signaling pathways were blocked; (g) the use of GR antagonists could increase the efficacy of the anti-melanoma immunotherapy. These facts highlight underlying mechanisms by which metastatic melanoma cells resist and adapt to survive. Consequently, it seems evident that elucidating the mechanisms involved in the pathophysiology of melanoma and the response to treatment is essential in advancing towards the development of personalized therapies.

## Data Availability

Not applicable.
